# Supplementary material for: In vivo Estimation of Axonal Morphology From Magnetic Resonance Imaging and Electroencephalography Data
Source: Front Neurosci. 2022 Apr 21;16:874023. doi: 10.3389/fnins.2022.874023 (PMC9070985; doi:10.3389/fnins.2022.874023)
Supplement: Supplementary file 1 [file Data_Sheet_1.DOCX]

Supplementary Material

1. Supplementary Figures


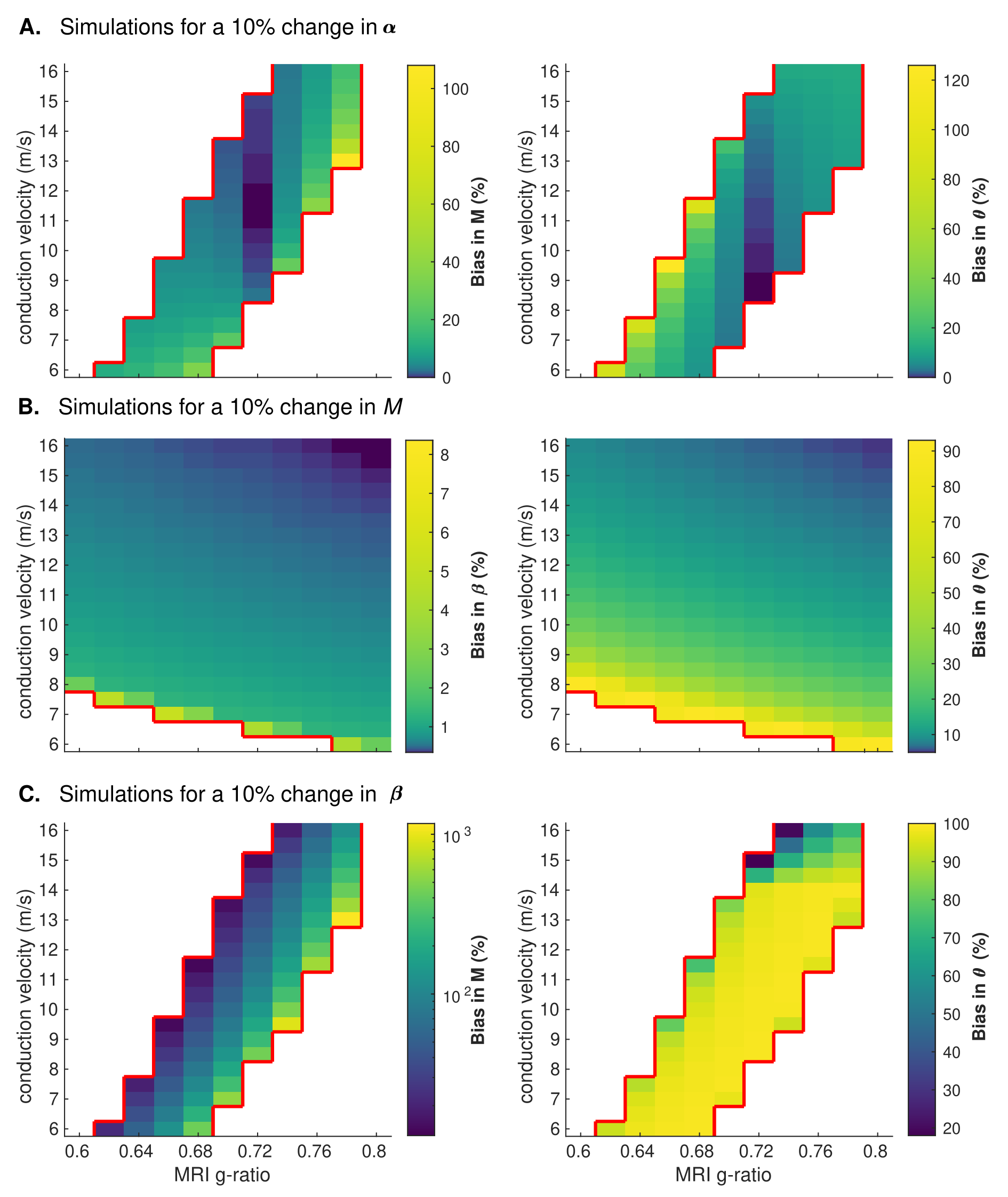


**Supplementary Figure 1.** Impact of inaccurate constant parameters on the estimated morphological features. (A) A 10% bias in α leads to an average bias of ~10% and ~15% for$M$ and $\theta$, respectively. For a representative combination of in-vivo parameter estimates ($g_{MRI}$=0.72, $V$=10 m/s), this bias is ~0.5% for $M$ and $\theta$. (B) A 10% bias of $M$ leads to an average bias of ~1% and ~22% for $\beta$ and $\theta$, respectively. For the representative combination of $g_{MRI}$ and $V$, this bias is <1% and ~16%. (C) A 10% bias of $\beta$ leads to an average bias of ~155% and ~92% for$M$ and $\theta$, respectively. For the representative combination of $g_{MRI}$ and $V$, this bias is ~145% for $M$ and ~100% for $\theta$. The red contour lines delineate the region of biologically plausible parameter values, compatible with the proposed model (Fig 3). Estimates outside this region were never observed in the in-vivo data and produced large values in these analyses and are therefore masked out.


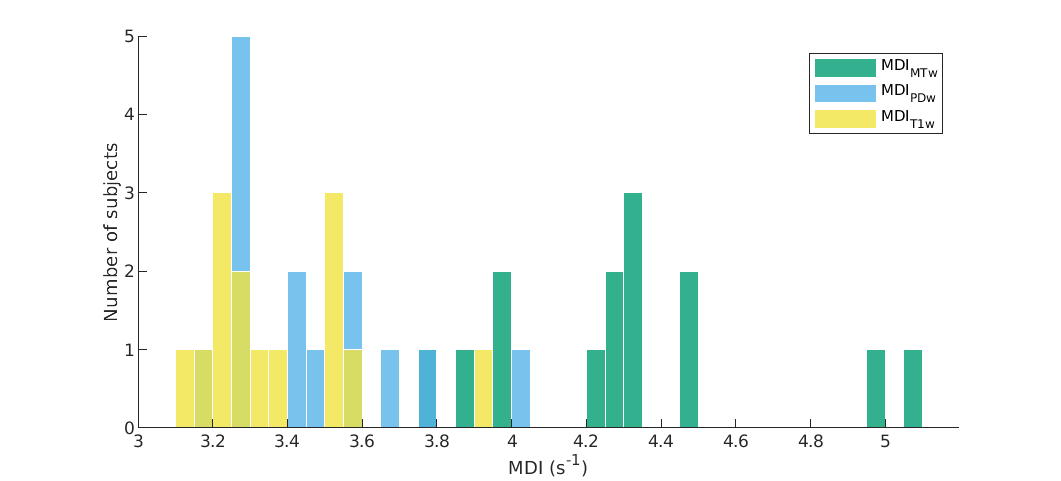


**Supplementary Figure 2.** Distribution of the Motion Degradation Index (MDI, s^-1^) values for the raw FLASH images across all subjects (N=14).


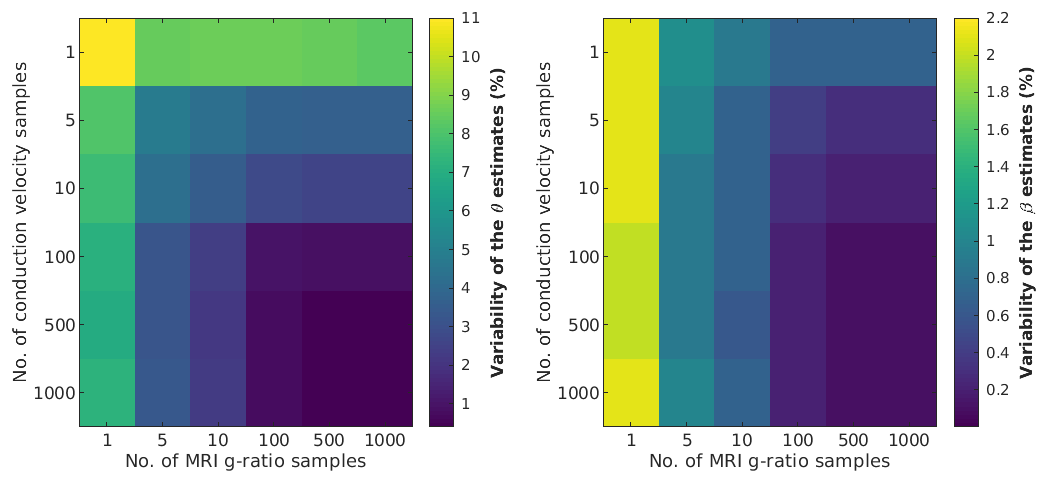


**Supplementary Figure 3.** Dependence of the variability of the $\theta$ (left) and $\beta$ (right) estimates on the number $g_{MRI}$ and $V$ samples. The variability of the $\theta$ estimates shows a strong dependence on the number of samples of both $V$ and $g_{MRI}$. The variability of$\beta$ is mostly driven by the number of $g_{MRI}$ samples.

1. Supplementary Appendix A

Our two morphological features of interest are defined as (see Theory section):

$$g(r)=\beta*r^{\alpha}$$

$$P\left( r \right)=P\left( r|k,\theta\right)=\frac{1}{\Gamma\left( k \right)\theta^{k}}r^{k-1}e^{\frac{-r}{\theta}}$$

With $k$ being the shape and$\theta$ the scale of the axonal radius distribution.

In agreement with West et al., 2016, the MRI g-ratio is written as an ensemble average of the axonal g-ratios within each image voxel, weighted by the axons’ cross-sectional area:

$$\begin{aligned} {g_{MRI}}^{2}= \frac{\int_{0}^{\infty} R^{2}{g\left( r \right)}^{2}P\left( r \right)dr}{\int_{0}^{\infty} R^{2}P\left( r \right)dr}= \frac{\int_{0}^{\infty} r^{2}P\left( r \right)dr}{\int_{0}^{\infty} \frac{r^{2}}{{g\left( r \right)}^{2}}P\left( r \right)dr} \#\left( 1 \right) \end{aligned}$$

where $r$ is the axon radius and $R$ the fiber radius ($R=r/g(r)$).

Using $P\left( r \right)$ and $g\left( r \right)$ as we defined previously, the integrand in the denominator can be re-written as a Gamma distribution with shape parameter $k'=k-2\alpha$, leading to:

$$\begin{aligned} {g_{MRI}}^{2}=\frac{\beta^{2}\int_{0}^{\infty} r^{2}P\left( r \right)dr}{\int_{0}^{\infty} r^{2-2a}P\left( r \right)dr}= \frac{\beta^{2}\int_{0}^{\infty} r^{2}\left( r \right)P\left( r|k,\theta\right)dr}{\frac{\Gamma\left( k^{'} \right)\theta^{k^{'}}}{\Gamma\left( k \right)\theta^{k}}\int_{0}^{\infty} r^{2}P\left( r|k^{'},\theta\right)dr} \#\left( 2 \right) \end{aligned}$$

These integrals can be transformed using the second moment of the radius distribution:

$$\begin{aligned} {g_{MRI}}^{2}= \frac{\beta^{2}\Gamma\left( k \right)\theta^{k}}{\Gamma\left( k^{'} \right)\theta^{k^{'}}}\frac{\left( k+1 \right)k\theta^{2}}{\left( k^{'}+1 \right)k^{'}\theta^{2}}= \frac{\beta^{2}\Gamma\left( k \right)\theta^{2\alpha}}{\Gamma\left( k^{'} \right)}\frac{\left( k+1 \right)k}{\left( k^{'}+1 \right)k^{'}} \#\left( 3 \right) \end{aligned}$$

For convenience, we make a change of variable, where $M=\theta(k-1)$, corresponding to the mode/peak of the radius distribution. Eq. (3) can be re-written as:

$$\begin{aligned} {g_{MRI}}^{2}=\beta^{2}\theta^{2\alpha}*\frac{\Gamma\left( \frac{M}{\theta}+1 \right)}{\Gamma\left( \frac{M}{\theta}+1-2\alpha\right)}\frac{M+3\theta+{2\theta^{2}}/M}{M+\left( 3-4\alpha\right)\theta+\left( 2+4\alpha^{2}-6\alpha\right){\theta^{2}}/M} \#\left( 4 \right) \end{aligned}$$

As described in Waxman and Bennett, 1972, axonal conduction velocity ($v$) can be derived from the morphological properties of axons using: $v [m/s]=p* \frac{d [\mu m]}{g}$, where $p$ (~5.5-6.0) represents the contribution of additional axonal factors to the propagation of action potentials (*e.g*. length of Ranvier nodes, electrical properties of the myelin membranes). Assuming an equal contribution from all axons to the conduction velocity *V* measured with EEG, we obtain:

$$\begin{aligned} V =5.5 \int_{0}^{\infty} \frac{2r P\left( r \right)}{g\left( r \right)} dr \#\left( 5 \right) \end{aligned}$$

Using $P\left( r \right)$ and $g\left( r \right)$ as we defined previously, the integrand in the denominator can be re-written as a Gamma distribution with shape parameter $k’’=k-\alpha$, leading to:

$$\begin{aligned} V =\frac{11}{\beta}\int_{0}^{\infty} r^{1-\alpha}P\left( r|k,\theta\right)dr =\frac{11}{\beta}\frac{\Gamma\left( k^{''} \right)\theta^{k^{''}}}{\Gamma\left( k \right)\theta^{k}}\int_{0}^{\infty} rP\left( r|k'',\theta\right)dr\#\left( 6 \right) \end{aligned}$$

These integrals can be transformed using the first moment of the radius distribution:

$$\begin{aligned} V =\frac{11}{\beta}\frac{\Gamma\left( k^{''} \right)\theta^{k^{''}}}{\Gamma\left( k \right)\theta^{k}}k^{''}\theta\#\left( 7 \right) \end{aligned}$$

This expression can be re-written as a function of $M$ and$\theta$:

$$\begin{aligned} V = \frac{11*\theta^{1-\alpha}}{\beta}\frac{\Gamma\left( \frac{M}{\theta}+1-\alpha\right)}{\Gamma\left( \frac{M}{\theta}+1 \right)}\left( \frac{M}{\theta}+1-\alpha\right) \#\left( 8 \right) \end{aligned}$$

Our final model incorporates Eqs. (4) and (8).
